# Free Carriers versus Self-Trapped Excitons at Different Facets of Ruddlesden–Popper Two-Dimensional Lead Halide Perovskite Single Crystals

**DOI:** 10.1021/acs.jpclett.1c01148

**Published:** 2021-05-20

**Authors:** Mingli Liang, Weihua Lin, Qian Zhao, Xianshao Zou, Zhenyun Lan, Jie Meng, Qi Shi, Ivano E. Castelli, Sophie E. Canton, Tönu Pullerits, Kaibo Zheng

**Affiliations:** † Department of Chemistry, Technical University of Denmark, DK-2800 Kongens Lyngby, Denmark; ‡ Chemical Physics and NanoLund, Lund University, Box 124, 22100 Lund, Sweden; # Department of Energy Conversion and Storage, Technical University of Denmark, DK-2800 Kongens Lyngby, Denmark; ∥ European XFEL, Holzkoppel 4, 22869 Schenefeld, Germany

## Abstract

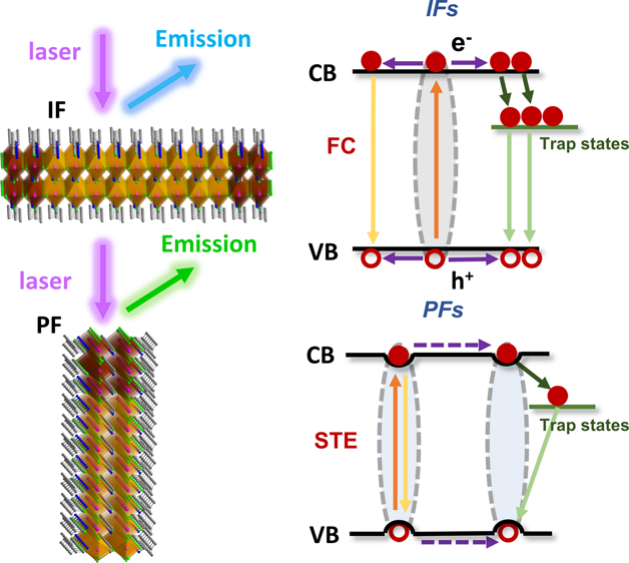

The physical origin of sub-band gap photoluminescence in Ruddlesden–Poppers two-dimensional (2D) lead halide perovskites (LHPs) is still under debate. In this paper, we studied the photoluminescence features from two different facets of 2D LHP single crystals: the in-plane facet (IF) containing the 2D inorganic layers and the facet perpendicular to the 2D layers (PF). At the IF, the free carriers (FCs) dominate due to the weak electron–phonon coupling in a symmetric lattice. At the PF, the strain accumulation along the 2D layers enhances the electron–phonon coupling and facilitates self-trapped exciton (STE) formation. The time-resolved PL studies indicate that free carriers (FCs) at the IF can move freely and display the trapping by the intrinsic defects. The STEs at the PF are not likely trapped by the defects due to the reduced mobility. However, with increasing STE density, the STE transport is promoted, enabling the trapping of STE by the intrinsic defects.

In Ruddlesden–Poppers (RP) two-dimensional lead halide perovskites (2D LHPs) (B)_2_(A)_*n*−1_Pb_*n*_X_3*n*+1_ (A = small amine cation, B = long chain organic amine cation, X = halides, *n* = number of octahedral layers), inorganic semiconductor quantum wells (QWs) with a certain thickness (*n*) are isolated periodically by organic spacing layers. Such a unique microscopic structure induces properties that differ from their 3D counterparts.^1−6^ Currently, one essential topic is to identify the nature of elementary photoexcitations. Wannier excitons are believed to be generated in semiconductor 2D quantum wells due to the dielectric confinement.^7^ 2D perovskites, on the other hand, possess a much softer lattice and more complex vibrational structures due to the intercalation of the organic spacing cations.^8^ Consequently, photoinduced local lattice distortion can occur due to strong electron–phonon coupling leading to the formation of self-trapped excitons (STEs).^9−12^ One fingerprint of such an STE is a broad sub-bandgap emission with a large Stokes shift.^13^ However, such emissions depend on the sample parameters such as *n* values and molecular composition.^14^ A recent photoluminescence (PL) microscopic study on single 2D perovskite flakes even disagrees with the assignment of the broad emission to STE.^15^ The photophysics in the 2D perovskites have also been reported as modulated by the local structures. Low-energy emission was observed specifically from the edge of single-crystal (SC) flakes interpreted by the formation of low energy edge states (LES).^16^ DFT calculations indicate that the formation of such LES is induced by the asymmetric relaxation of the interface strain that triggers the surface reorganization.^17,18^ In this regard, the photogenerated species in the bulk volume and at the edge area can be different due to the difference in local electronic structures. Therefore, identifying the photogenerated species and clarifying the corresponding dynamics within various local structures is essential to characterize the actual photophysics in 2D perovskite materials.

In this paper, we investigate the emissive state dynamics to identify the photogenerated species at two different surface facets of 2D RP lead bromide perovskite corresponding to in-plane facets (IFs) and perpendicular facets (PFs) to the 2D inorganic lattice layers, respectively. Single crystals with three spacing cations [i.e., iso-butylamine (iso-BA), *n*-butylamine (*n*-BA), and *n*-pentylamine (*n*-PA)] have been studied. The temperature-dependent PL measurements indicate that STEs are dominant at the PF while free carriers (FC) are generated at the IF. The STE formation is strongly affected by the spacing cations. Furthermore, the time-resolved PL study reveals that the STEs at the PFs are less mobile than the FCs at the IFs and exempt from the intrinsic defect state trapping. However, when the excitation density is increased, the large STE concentration facilitates the STE transport. The STEs are therefore more likely to be captured by the intrinsic trap states. Our results confirmed the photogenerated species strongly depend on the local structures in 2D perovskites which leads to distinct photophysical behaviors.

According to the DFT calculation, the LES in 2D RP perovskites are mainly induced by the relaxation of the interface strain along the 2D inorganic layer nucleating the surface reorganization, while such strain along the direction perpendicular to the 2D layer can be released by the spacing cations.^17^ Here, as demonstrated by an example structure of as-obtained (B)_2_(A)_*n*−1_Pb_*n*_X_3*n*+1_ (*n* = 2) (Figure 1), the PbX_6_ octahedra are extended by the cocorner links along the (200) facet forming the largest crystal facet in the macroscopic crystal. The crystals grow slowly along the (002), (110), and (111) facets due to the superimposed organic spacing layers and the inorganic QWs leading to small crystal facets (Figure 1a,b). The lattice strain along the direction vertical to the (200) facet can be released into the organic spacing cations (Figure 1c). Thus, we can consider the (200) surface facet identical to a bulk-like structure. On the other hand, the lattice strain continuously accumulates along the (002), (110), and (111) directions and releases at the surface boundary. Therefore, the surface lattice structure along these directions should be different from the inner bulk structure, resulting in the so-called edge area where LES are found (Figure 1c).^16^

**Figure 1:**
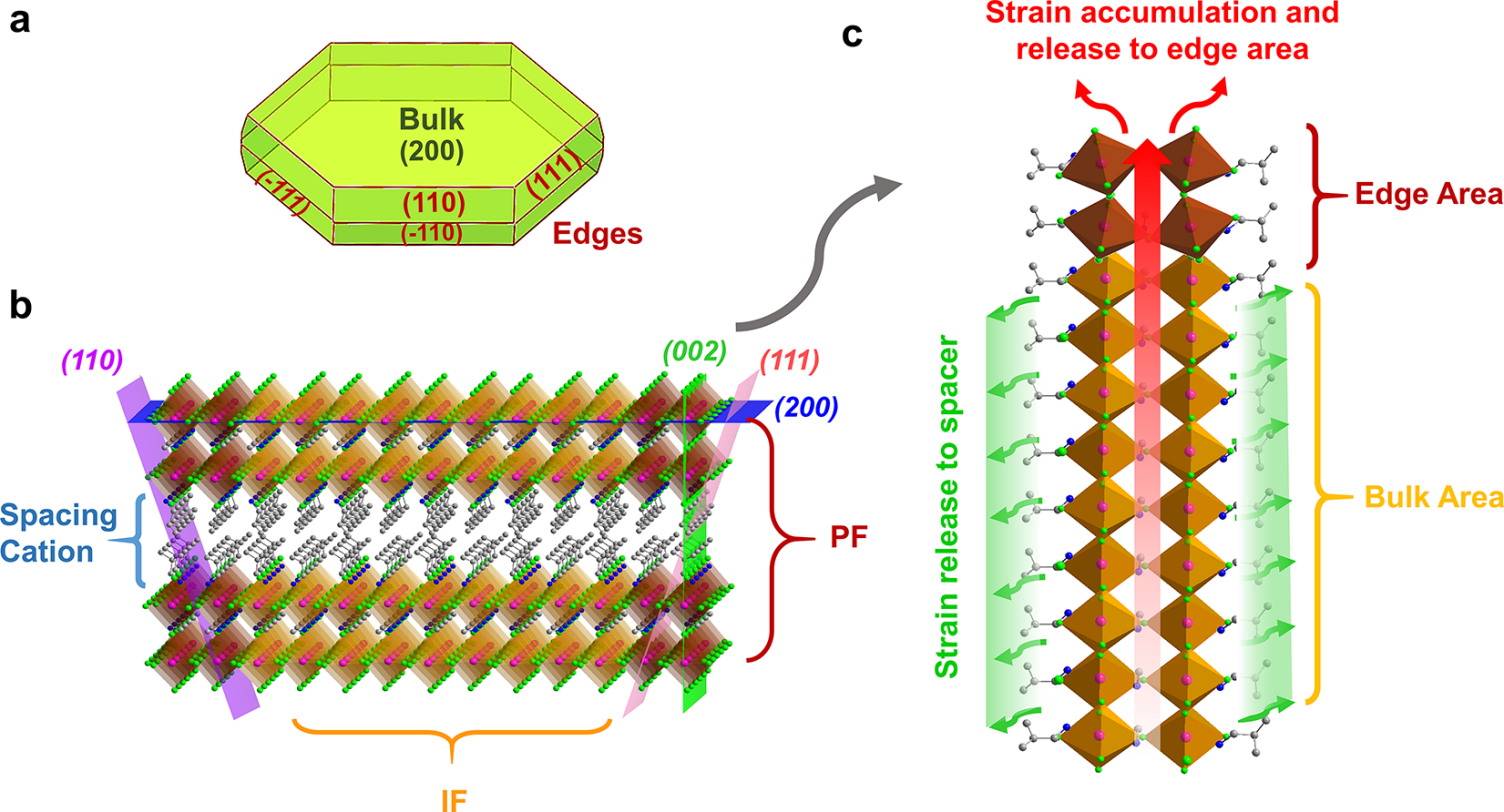
Macroscopic morphology of a 2D RP perovskite crystal (a), which is simulated by Mercury based on our structural data in the Supporting Information; schematics showing the definition of IF and PF (b) and the directions of strain accumulation release (c).

Since the LES formation is induced by the accumulation and relaxation of strain-related elastic energy, spacing cations are expected to play a critical role in modulating the LES.^19,20^ Therefore, by changing the spacing cations, we can expect to facilitate or diminish the LES formation. In this work, we have synthesized three 2D perovskites SCs with various spacing cations, (iso-BA)_2_(MA)Pb_2_Br_7_ (iso-BAPB), (*n*-BA)_2_(MA)Pb_2_Br_7_ (*n*-BAPB), and (*n*-PA)_2_(MA)Pb_2_Br_7_ (*n*-PAPB) (for experimental details and characterizations, see Supporting Information Section S1). They all belong to the typical 2D RP phase perovskites (B)_2_(A)_*n*−1_Pb_n_X_3*n*+1_ with *n* = 2. The powder XRD patterns of the crystal in Figure 2a all exhibited perfect 2D layered structure as illustrated in Figure 2b–d. Crystallographic analysis shows that the lattice of *n*-BAPB is the least distorted while storing the strain-related elastic energy to the largest extent, whereas that of *n*-PAPB is the most distorted when relaxing the interface lattice strain (for the details of the structural analysis, see Supporting Information Section S2). In order to evaluate the strain accumulation along the 2D layered facets for three SCs, we calculated the internal lattice mismatch by simplfying the 2D structures as the bulk 3D lattice connected to a 2D LHP layer at the interface of the quantum wells (Figure 2e, for the details of mismatch caculation, see Supporting Information Section S3).^21,22^ As shown in Figure 2f, the internal lattice mismatch of *n*-PAPB is much smaller than those of the other two samples, leading to a smaller elastic energy and consequently less pronounced LES formation on the PFs.

**Figure 2:**
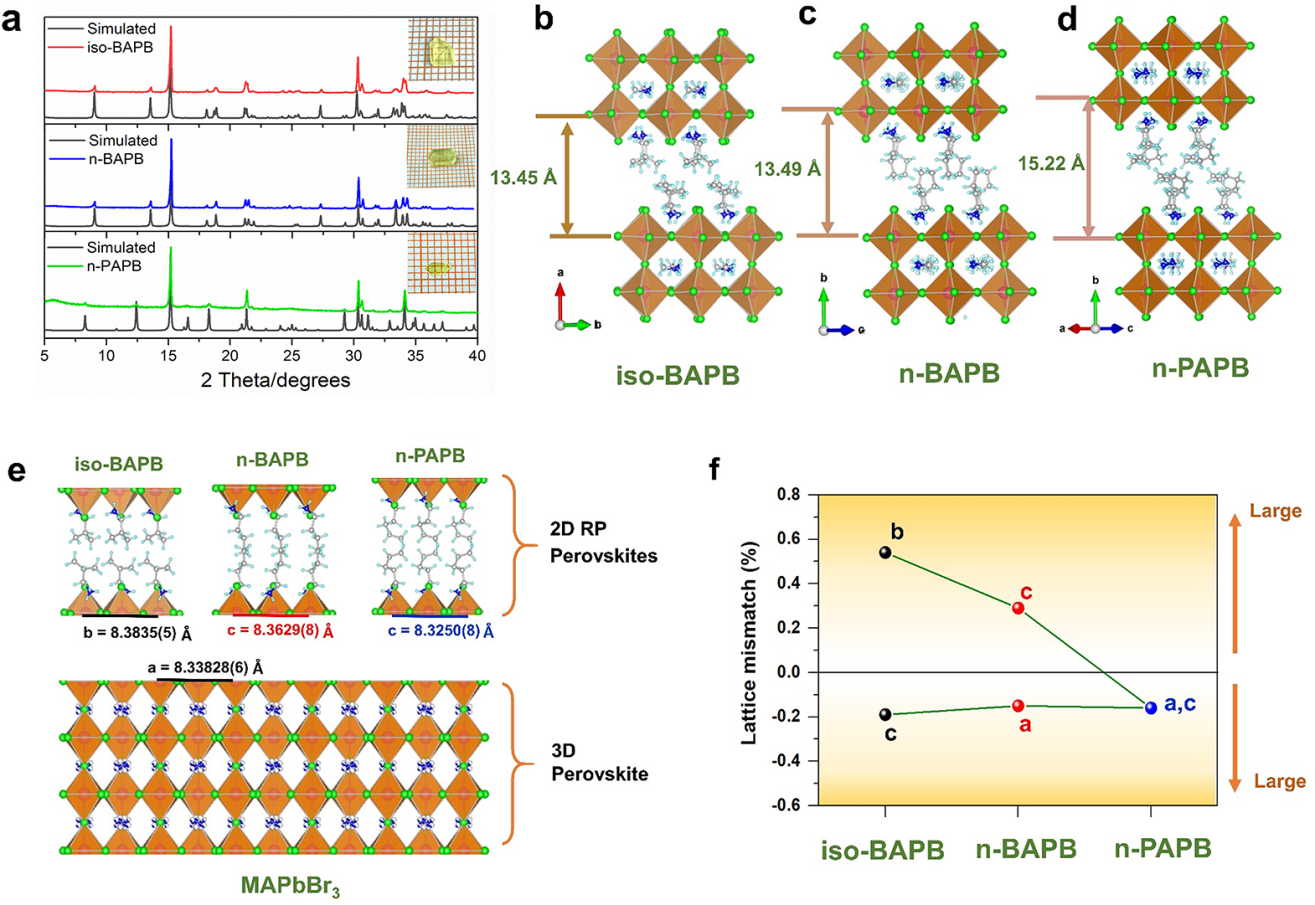
Powder XRD patterns of iso-BAPB, *n*-BAPB, and *n*-PAPB SCs (inset: optical pictures of the three SCs with the size of each red grid in the background being 1 mm × 1 mm) (a); 2D layered structures of iso-BAPB (b), *n*-BAPB (c), and *n*-PAPB (d); the changes of the lattice constants on the 2D layered facets compared to that of 3D MAPbBr_3_ (*I*4/*mcm*) (e); lattice mismatch between the three 2D RP perovskites and the 3D MAPbBr_3_ lattice; and the two points of each sample representing two directions on the 2D layered facets (f).

Afterward, we measure the steady-state PL spectra at both PF and IF of the three SCs as discussed above (Figure 3a). The configuration of the PL measurement ensures the signals are solely contributed by the incident beam area at the surface of each facet (for the details of the method, see Supporting Information Sections S1 and S4). As shown in Figure 3b, the emissions from different facets in iso-BAPB and *n*-BAPB exhibit clear single bands, but the PL at the PF drastically red shifts compared to that of the IF by 85 nm (∼0.45 eV) and 82 nm (∼0.43 eV), respectively. In *n*-PAPB, two emission peaks occur at the PF, while the low-energy emission is less red-shifted (75 nm, ∼0.41 eV) compared with the other two samples.

**Figure 3:**
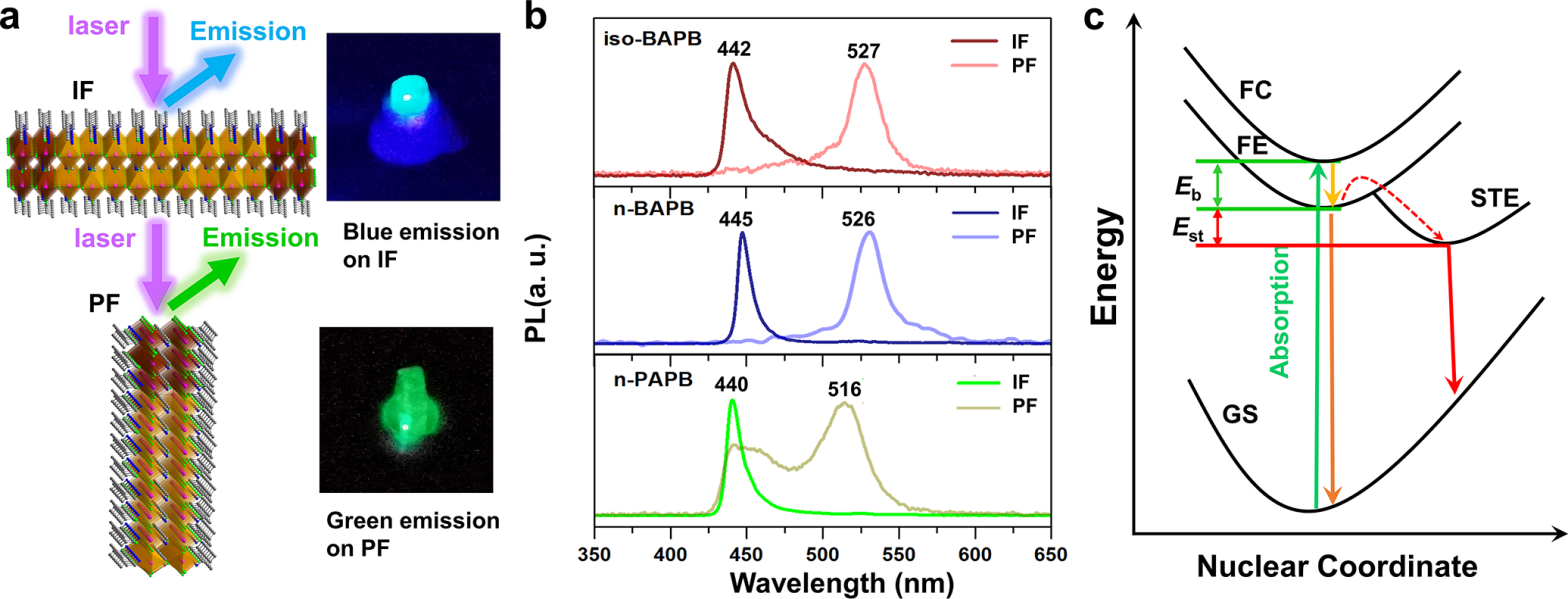
Schematics of PL measurements and the pictures of blue emission on IF and green emission on PF of the SC (a); PL spectra of PF and IF of the three SCs (b) and the schematic of the energy level structure of STE (c) (FC is the free carrier state; FE is the free exciton state; GS is the ground state; *E*_st_ is self-trapping energy).

The difference in the PL emissions at the two facets cannot be merely explained by static lattice distortion since the energy shift (i.e., 0.4–0.45 eV) is much larger than what the octahedral distortion-induced bandgap modification can provide.^23^ The origin of the low-energy emission in 2D RP LHPs which is especially pronounced at the “edge” of the grains or crystals has been frequently debated in recent years. We can first exclude the formation of the 3D phase claimed in some recent studies since no 3D phase can be observed in the powder XRD characterization of the ground crystals (Figure S1).^24,25^ In the following, we will also exhibit that the photophysics at the PFs are different from that in conventional bulk 3D perovskites. The red-shifted emission is usually assigned to STEs. Figure 3c is a schematic of the energy level structure of STE, in which, once electrons and holes are photogenerated, they will quickly self-trap from a mobile state to a more stable self-trapped state. Here the self-trapping energy (*E*_st_) is defined as the energy loss of exciton in this process.^12^ However, recent PL microscopy studies argued that the low-energy emission in 2D RP LHPs should be more related to the mid-band gap trap states trapping the diffusing FEs (free excitons).^15^

In order to clarify the physical process, we conducted temperature-dependent PL measurements at the IFs and PFs with temperatures from 100 to 280 K for three samples (Figure S7). For *n*-PAPB, the PL intensity of the IF decreases with increasing temperature due to the dissociation of the excitons into free charges. On the other hand, the dual emission peaks occur all over the temperature region at the PF, whereas the respective fraction of high-energy PL becomes more dominant at low temperature. This phenomenon is against the traditional exciton trapping mechanism where the emissions from the trap state and band-edge exciton states are competing and modulated by the thermal equilibrium between the trapping and detrapping of the excited carriers. Therefore, we believe the emission at the PF should be attributed to STEs formed by Fröhlich interaction as shown in Figure 3c, which decreases at low temperature.^26^ On the other hand, at lower temperature the energy barrier between the FE and the STE states will hinder the self-trapping and promote the band-edge emission (Figure 3c). The PL spectra at PF of *n*-BAPB and iso-BAPB only contain the low-energy emission with the absence of band-edge emission regardless of the temperature, indicating no deactivation from STE to FE in those samples (i.e., larger *E*_st_).

In order to further verify our argument, we calculate the electron–phonon coupling strength from the fwhm’s of the temperature-dependent PL spectra using the model in refs (26 and 27), and the line width parameters are shown in Table 1 (for the details of the analysis, see Supporting Information Section S5). The fitted values of energy representative of the frequency for the weakly dispersive LO phonon branch (*E*_LO_) are much larger than those of the vibration mode of Pb–Br stretching (18 meV), which is believed to dominate the Fröhlich interaction in 3D perovskites.^26,28^ In 2D RP perovskites, however, *E*_LO_ only provides an effective value, which should not be associated with specific phonon modes due to the complex vibrational structure.^29−31^ The vibrational modes of tens of millielectronvolts usually correspond to the rotation or bending of large molecular moieties (e.g., NH_3_^+^).^9^ More importantly, the coupling strength γ_LO_ at the PFs are larger than those at the IFs in three samples except for *n*-PAPB. The stronger PF electron–phonon (e-ph) coupling drives the formation of STE in our 2D perovskite SCs. In addition, the relatively weaker coupling of excitons or charge carriers with lattice deformation at the PF of *n*-PAPB explains the less stabilized STE as mentioned above. We noticed that the bandwidth of such STE emission is still much narrower than conventional observation in 2D perovskites or other polaronic semiconductors.^32,33^ The narrowing of the STE emission here can be due to two factors: (1) relative weaker e–ph coupling at the PF of our SCs compared with traditional polaronic materials and/or (2) that the vibration energy of the above-mentioned large molecular moieties that contributes to the e–ph coupling should be less varied by the surrounding coordination compared with Pb–Br stretching vibration.

**Table 1:** Extracted Line Width Parameters from the Temperature Dependent PL Measurement

IFs	Γ_0_ (meV)	γ_LO_ (meV)	*E*_LO_ (meV)
iso-BAPB	69.1	251.5	49.5
*n*-BAPB	61.3	359.9	45.1
*n*-PAPB	56.1	218.3	50.3

PFs	Γ_0_ (meV)	γ_LO_ (meV)	*E*_LO_ (meV)
iso-BAPB	94.8	452.5	63.1
*n*-BAPB	101.1	434.1	60.3
*n*-PAPB	127.5	233.4	49.6

In order to further distinguish the photophysics at the IFs and PFs, we studied the PL dynamics at the two facets of the three samples (Figure 4a–f) with the corresponding excited state dynamic demonstrated in Figure 4g–i. Figure 4a shows the PL lifetime at the PF is longer than at the IF (for detailed fitting analysis of the PL decays, see Supporting Information Section S6). In addition, the PL lifetimes of the in-plane state increase with the excitation intensity as illustrated in Figure 4b. This behavior has been explained by trap filling/accumulation (Figure 4g), which is widely observed in 2D and 3D perovskite single and microcrystals.^34,35^ We can obtain trap density of the IFs to be 2.9 × 10^16^ cm^–3^ by globally fitting the intensity-dependent PL kinetics using a dynamic trap-filling model (for the details of the model, see Supporting Information Section S7).^36^ More importantly, such behavior indicates that the initial photogenerated species at the IFs are free carriers moving along the perovskite lattices until filling a trap. In fact, we can estimate the fraction of free carriers in the photogenerated species using the Saha–Langmuir theory between 84% to 99% at the IFs of our samples (for details, see Supporting Information Section S8). On the contrary, the PL decay becomes faster with the increasing excitation intensity at the PFs, indicating a different charge recombination process (Figure 4c). We can first exclude the occurrence of any high order recombination because the intensity of the PL decay at time zero (PL_0_) exhibits a linear dependence on the excitation intensity, as shown in the inset pictures of Figure 4d–f. As PL_0_ reflected the population of all the spontaneous emission before any trapping or the Auger process, such linearity manifests the monomolecular recombination mode of the photoexcited species.^37^ The deviation of the intensity-dependent PL kinetics at the PF can be well interpreted by the STE model. Unlike in 3D perovskite or IFs in 2D perovskite where the electron–phonon coupling generates mainly a large polaron, a larger degree of lattice distortion at the PFs of 2D perovskites tends to form small polarons that move through site-to-site hopping.^36,38^ The intrinsic trap density of the *n*-BAPB SCs calculated above provides a mean interval distance among the trap states to be 32 nm. It is difficult for the STE at the PFs to transport to the trap state at low excitation density and undergo a trapping process due to the reduced mobility after self-trapping. Therefore, the dominant excited state depopulation pathway is the STE radiative recombination as shown in Figure 4h. With the increase of the excitation intensity, the relative PLQY at the PFs also decreases, indicating that extra carrier trapping has been introduced at a high excitation density (Table S8 and Figure S10). We believe it can be attributed to the enhanced motion of the STEs to be trapped by the defect states as illustrated in Figure 4i. Two possible interpretations can be provided: (1) The stability of the STE can be decreased by the increasing density.^39^ Therefore, more STEs can be dissociated into FEs or free carriers. (2) A larger STE density may increase the carrier mobility due to the collective polaron behavior, which is widely observed in OPV materials and graphene.^40,41^ In iso-BAPB, however, the PL decay remains independent of excitation intensity. One possible reason is the larger trap density in iso-BAPB SCs (for a detailed calculation, see Supporting Information Section S7). In addition, we noticed the efficient exciton dissociation has been reported at the edge area for *n*-BA based 2D RP iodide perovskites,^6,9,42^ which deviates from our conclusion. We believe this is due to the higher bulk modulus and larger lattice polarity of the Br-based perovskites that enhanced the local strain accumulation and phonon coupling, respectively (for detailed analysis, see Supporting Information Section S9). Both factors facilitate the STE formation.

**Figure 4:**
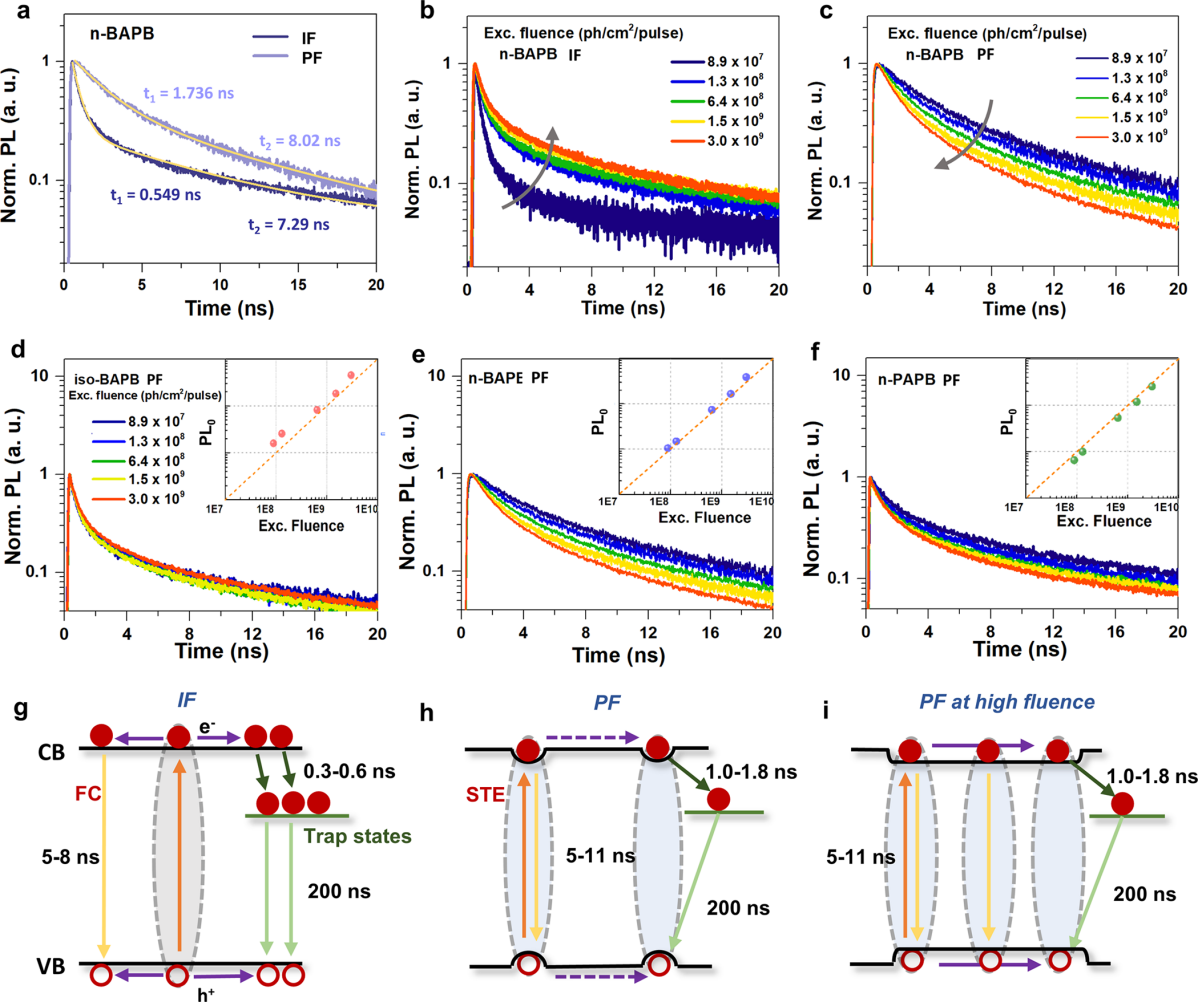
TRPL kinetics of IFs and PFs of *n*-BAPB (a–c) and TRPL kinetics with different excitation fluences for the PFs of these three SCs (d–f). Insets: initial PL intensity change with fluence. Illustrations of the charge recombination processes in these three SCs (g–i).

In conclusion, we investigated the local structure and PL dynamics at different surface facets (IF and PF) of 2D RP lead bromide perovskite SCs with three spacing cations (i.e., *n*-BA, iso-BA, and *n*-PA). The PL spectra at the IF of three SCs exhibit typical pure band-edge emission bands, while the emission at the PF is drastically red-shifted. In addition, at the PF of *n*-PAPB, the band edge emission and the red-shifted emission occur concurrently. The temperature-dependent PL study at two facets indicates that the low-energy emission at PF should be attributed to the STE due to larger electron–LO phonon coupling strength compared with IFs. The dual emission bands in *n*-PAPB can be explained by the lower self-trapping energy, which is consistent with the structural analysis. In addition, we find the PL decays become slower with the increasing excitation density at the IFs with opposite behavior at PFs. This can be well interpreted by the different photophysics of photogenerated FCs at the IF and STEs at the PF. Our findings confirmed the origin of the LES in 2D RP perovskites to be the STE. The STE formation is also strongly influenced by the spacing cations in the 2D lattice. That conclusion can guide materials engineering and device applications in the future.

## Supporting Information Available

Experimental details and characterizations, crystallographic data, values of Pb–Br bond distances and Br–Pb–Br bond angles, TRPL kinetic study, details about calculations of lattice distortion parameters, lattice mismatch, penetration depths, electron–phonon coupling strength, and trap densities (PDF)
Crystallographic information file for iso-BAPB (CIF)
Crystallographic information file for *n*-BAPB (CIF)
Crystallographic information file for *n*-PAPB (CIF)

## Supplementary Material

jz1c01148_si_001.pdf

jz1c01148_si_003.cif

jz1c01148_si_004.cif

jz1c01148_si_005.cif
