# Supplementary material for: Free Carriers versus Self-Trapped Excitons at Different Facets of Ruddlesden–Popper Two-Dimensional Lead Halide Perovskite Single Crystals
Source: J Phys Chem Lett. 2021 May 20;12(20):4965–71. doi: 10.1021/acs.jpclett.1c01148 (PMC8279734; doi:10.1021/acs.jpclett.1c01148)
Supplement: Supplementary file 1 — jz1c01148_si_001.pdf [file jz1c01148_si_001.pdf]

Free Carriers versus Self-Trapped Excitons at Different Facets of Ruddlesden-Popper Two-Dimensional Lead Halide Perovskite Single Crystals

Mingli Liang[†], Weihua Lin[‡], Qian Zhao[†], Xianshao Zou[‡], Zhenyun Lan[#], Jie Meng[†], Qi Shi[‡], Ivano E. Castelli[#], Sophie E. Canton[^], Tönu Pullerits[‡] and Kaibo Zheng[*,†, ‡]

[†]*Department of Chemistry, Technical University of Denmark, DK-2800 Kongens Lyngby, Denmark;*

[‡]*Chemical Physics and NanoLund, Lund University, Box 124, 22100 Lund, Sweden;*

[#]*Department of Energy Conversion and Storage, Technical University of Denmark, DK-2800 Kongens Lyngby, Denmark;*

[^]*European XFEL, Holzkoppel 4, 22869 Schenefeld, Germany*

[*]Corresponding Author

Kaibo Zheng: kzheng@kemi.dtu.dk

Supporting Information

S1 Experimental details and characterizations

Materials: $PbBr_2$ (98 %), methenamine hydrobromide (98 %, MABr), iso-butylamine (99 %, iso-BA), *n*-butylamine (99.5 %, *n*-BA), *n*-pentylamine (99.5 %, *n*-PA), ethanol (99.8%) and hydrobromic acid (HBr, 47 % in water) have been utilized without further treatment. All the chemicals were supplied by Sigma-Aldrich.

Synthesis of long-chain amine bromides: The long-chain amine bromides were manufactured by adding equal stoichiometric ratio of iso-BA, *n*-BA and *n*-PA to dilute aqueous HBr (25% in mixed ethanol and water) under vigorous stirring in an ice-water bath. The white products were acquired by rotary evaporation at 60 ℃. After

that, a lot of white crystals of long-chain amine bromides (iso-BABr, *n*-BABr, and *n*-PABr) were obtained by washing three times with ethanol and vacuum drying for 24h.

Synthesis of 2D RPP SCs: $(iso\text{-}BA)_2(MA)Pb_2Br_7$ (iso-BAPB), $(n\text{-}BA)_2(MA)Pb_2Br_7$ (*n*-BAPB) and $(n\text{-}PA)_2(MA)Pb_2Br_7$ (*n*-PAPB) SCs obtained by the temperature lowering method of $PbBr_2$ (2.5 mmol), MABr (1.5 mmol), iso-BABr/ *n*-BABr/ *n*-PABr (3.0 mmol) in 5 ml HBr (47 % in water). The mixtures were placed into glass bottles (20 ml). Afterwards, the glass bottles were sealed and stirred at room temperature for 30 minutes to induce yellow precipitates. Completely clear solutions were obtained after reaction for few minutes at 80 °C as a precursor. Bulk SCs were grown from such a solution at a cooling rate of 0.5 °C/day starting from 50 °C.

Characterizations: Single-crystal XRD measurements were performed on SuperNova Dual Wavelength CCD diffractometer (Agilent Technologies, Mo-Kα with λ = 0.71073 Å) at room temperature (~298 K). Further structural solves and refinements by full-matrix least-squares fitting on F^2 using SHELX-97, the details are similar to our recent work.[1] Crystallographic data and structural refinements for three samples are summarized in Table S1. The Pb-Br bond lengths and Br-Pb-Br angles are listed in Table S2 and S3, respectively. Powder XRD data were collected on a Rigaku MiniFlex 136 II diffractometer using Cu-Kα radiation with λ = 1.5406 Å. In addition, our comparison with 3D $MAPbBr_3$ perovskite XRD data shows that our samples are pure phases with the absence of 3D $MAPbBr_3$ impurity (Figure S1). The UV−vis absorption spectra were measured on PerkinElmer (Lambda 1050) with a UV-Vis-NIR absorption spectrophotometer. Steady-state PL spectra were acquired on an Avantes AvaSpec-2048 spectrometer under an excitation wavelength of 375 nm. The time-resolved photoluminescence (TRPL) measurements were performed with time-correlated single-photon counting (TCSPC, Picoharp) by using a pulsed diode laser (Wavelength: 375 nm; Frequency: 2.5 MHz; Pulse duration: 8 ps) and a fast avalanche photodiode; when measuring TRPL spectra of IFs and PFs, a long-pass filter from 400 nm and a long-pass filter from 470 nm are used, respectively. Figure S2 are the brief set-up schematics of the steady-state PL and the TCSPC spectroscopy. The beam size (~0.12 mm^2) is much smaller than the area of each faceted surface, ensuring that the PL signals are solely contributed by the incident beam area**.** During their measurements, we adjusted the angle of the crystal so that the laser could be incident perpendicularly to the different facets.

Table S1. Crystal data and structure determinations of the iso-BAPB, *n*-BAPB and *n*-PAPB.

Samples	iso-BAPB	*n*-BAPB	*n*-PAPB
Formula Weight	1154.11	1154.11	1199.20
Space group	*Cc*	*Ccc2*	*Ccc2*
a (Å)	39.173(4)	8.3262(7)	8.3250(6)
b (Å)	8.3835(5)	39.247(4)	42.717(4)
c (Å)	8.3225(5)	8.3629(8)	8.3250(8)
α (deg)	90	90	90
β (deg)	90.789(7)	90	90
γ (deg)	90	90	90
V (Å^3)	2732.9(4)	2732.8(5)	2960.5(4)
Z	4	4	4
μ(Mo Ka) (mm^{-1})	22.537	22.538	20.811
GOF on F^2	1.031	1.065	1.046
R_1,wR_2 [I>2σ(I)]^a	0.0892, 0.2080	0.0789, 0.1935	0.0633, 0.1406
R_1, wR_2 (all data)^a	0.1300, 0.2587	0.1235, 0.2231	0.1241, 0.1707

$^aR_1 = \sum||F_o| - |F_c||/\sum|F_o|$, $wR_2 = \{\sum w[(F_o)^2 - (F_c)^2]^2/\sum w[(F_o)^2]^2\}^{1/2}$

Table S2. Bond distances (Angstroms) of Pb-Br for the iso-BAPB, *n*-BAPB and *n*-PAPB.

iso-BAPB			
Pb(1)-Br(4)	2.884(5)	Pb(2)-Br(10)	2.951(5)
Pb(1)-Br(9)	2.979(3)	Pb(2)-Br(7)#1	2.981(3)
Pb(1)-Br(9)#1	2.988(3)	Pb(2)-Br(7)	2.992(3)
Pb(1)-Br(3)	3.009(3)	Pb(2)-Br(6)	3.017(3)
Pb(1)-Br(3)#2	3.016(3)	Pb(2)-Br(6)#2	3.025(3)
Pb(1)-Br(5)	3.061(9)	Pb(2)-Br(5)	3.071(9)
n-BAPB			
Pb(1)-Br(6)	2.908(4)	Pb(1)-Br(3)	3.002(8)
Pb(1)-Br(4)	2.968(9)	Pb(1)-Br(3)#2	3.004(6)
Pb(1)-Br(4)#1	2.980(7)	Pb(1)-Br(2)	3.0636(9)
n-PAPB			
Pb(1)-Br(4)	2.890(3)	Pb(1)-Br(3)#2	2.998(6)

Pb(1)-Br(3)	2.925(7)	Pb(1)-Br(5)	3.034(5)
Pb(1)-Br(5)#1	2.967(4)	Pb(1)-Br(2)	3.0697(8)

Symmetry transformations used to generate equivalent atoms:

For iso-BAPB: #1 x,-y+1,z+1/2; #2 x,-y+2,z+1/2

For n-BAPB: #1 -x,y,z+1/2; #2 -x+1,y,z-1/2

For n-PAPB: #1 -x,y,z+1/2; #2 -x+1,y,z-1/2

Table S3. Br-Pb-Br angles (deg) for the iso-BAPB, *n*-BAPB and *n*-PAPB.

iso-BAPB			
Br(4)-Pb(1)-Br(9)	90.09(13)	Br(10)-Pb(2)-Br(7)#1	89.54(12)
Br(4)-Pb(1)-Br(9)#1	93.44(13)	Br(10)-Pb(2)-Br(7)	95.31(12)
Br(9)-Pb(1)-Br(9)#1	90.29(3)	Br(7)#1-Pb(2)-Br(7)	90.46(3)
Br(4)-Pb(1)-Br(3)	86.23(12)	Br(10)-Pb(2)-Br(6)	92.08(12)
Br(9)-Pb(1)-Br(3)	89.45(9)	Br(7)#1-Pb(2)-Br(6)	177.21(12)
Br(9)#1-Pb(1)-Br(3)	179.58(13)	Br(7)-Pb(2)-Br(6)	91.65(8)
Br(4)-Pb(1)-Br(3)#2	89.41(12)	Br(10)-Pb(2)-Br(6)#2	86.53(11)
Br(9)-Pb(1)-Br(3)#2	178.21(9)	Br(7)#1-Pb(2)-Br(6)#2	89.15(8)
Br(9)#1-Pb(1)-Br(3)#2	91.45(9)	Br(7)-Pb(2)-Br(6)#2	178.11(14)
Br(3)-Pb(1)-Br(3)#2	88.81(3)	Br(6)-Pb(2)-Br(6)#2	88.68(3)
Br(4)-Pb(1)-Br(5)	174.77(9)	Br(10)-Pb(2)-Br(5)	173.14(13)
Br(9)-Pb(1)-Br(5)	92.33(16)	Br(7)#1-Pb(2)-Br(5)	89.24(16)
Br(9)#1-Pb(1)-Br(5)	91.18(13)	Br(7)-Pb(2)-Br(5)	91.45(13)
Br(3)-Pb(1)-Br(5)	89.16(12)	Br(6)-Pb(2)-Br(5)	88.88(15)
Br(3)#2-Pb(1)-Br(5)	88.03(15)	Br(6)#2-Pb(2)-Br(5)	86.70(13)
n-BAPB			
Br(6)-Pb(1)-Br(4)	91.2(3)	Br(4)#1-Pb(1)-Br(3)#2	176.2(3)
Br(6)-Pb(1)-Br(4)#1	90.4(2)	Br(3)-Pb(1)-Br(3)#2	90.12(6)
Br(4)-Pb(1)-Br(4)#1	90.36(5)	Br(6)-Pb(1)-Br(2)	179.4(6)
Br(6)-Pb(1)-Br(3)	91.2(3)	Br(4)-Pb(1)-Br(2)	89.3(3)
Br(4)-Pb(1)-Br(3)	176.3(2)	Br(4)#1-Pb(1)-Br(2)	89.2(2)
Br(4)#1-Pb(1)-Br(3)	86.9(3)	Br(3)-Pb(1)-Br(2)	88.2(3)
Br(6)-Pb(1)-Br(3)#2	92.0(2)	Br(3)#2-Pb(1)-Br(2)	88.3(2)
Br(4)-Pb(1)-Br(3)#2	92.5(3)		

n-PAPB			
Br(4)-Pb(1)-Br(3)	91.3(3)	Br(5)#1-Pb(1)-Br(5)	89.68(4)
Br(4)-Pb(1)-Br(5)#1	90.1(2)	Br(3)#2-Pb(1)-Br(5)	85.6(3)
Br(3)-Pb(1)-Br(5)#1	94.5(3)	Br(4)-Pb(1)-Br(2)	179.1(4)
Br(4)-Pb(1)-Br(3)#2	90.8(2)	Br(3)-Pb(1)-Br(2)	88.6(3)
Br(3)-Pb(1)-Br(3)#2	90.22(4)	Br(5)#1-Pb(1)-Br(2)	89.0(2)
Br(5)#1-Pb(1)-Br(3)#2	175.2(3)	Br(3)#2-Pb(1)-Br(2)	90.1(2)
Br(4)-Pb(1)-Br(5)	89.7(3)	Br(5)-Pb(1)-Br(2)	90.5(3)
Br(3)-Pb(1)-Br(5)	175.7(2)		

Symmetry transformations used to generate equivalent atoms:

For iso-BAPB: #1 x,-y+1,z+1/2; #2 x,-y+2,z+1/2

For n-BAPB: #1 -x,y,z+1/2; #2 -x+1,y,z-1/2

For n-PAPB: #1 -x,y,z+1/2; #2 -x+1,y,z-1/2

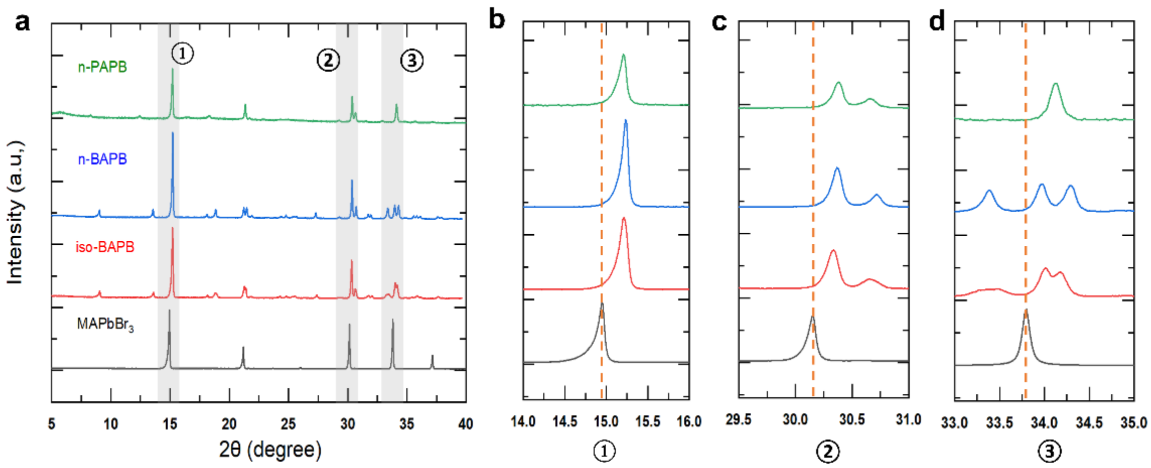

Figure S1. Comparison of powder XRD patterns of our three samples and pure $MAPbBr_3$ (a); the magnified area corresponding to the three strong peaks (b-d).

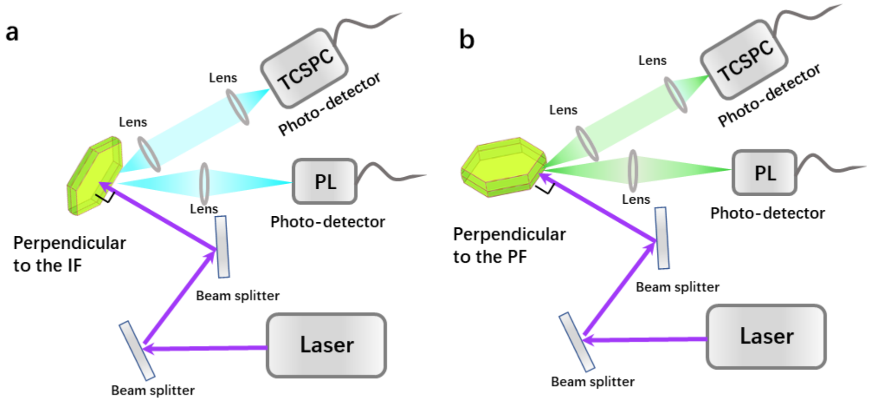

Figure S2. The set-up schematics of PL and TCSPC measurements on IF (a) and PF (b).

S2: Calculations of tilting distortion parameter (Δ), the angle distortion parameter (Σ), and the off-center distortion parameter (Δ_{od}).

When the lone-pair cations (e.g., Sn^{2+}, Pb^{2+}, Bi^{3+}, etc.) and d^0 transition metals (e.g., Nb^{5+}, V^{5+}, Mo^{6+}, etc.) form a polyhedron with oxygen or halide ions, the primary distortive cause can be attributed to second-order Jahn-Teller (SOJT) effects (electronic effects).[2,3] For instance, Figure S3 is a $PbBr_6$ octahedron from the iso-BAPB sample of this work, in which the Pb^{2+} deviate from the center position where they should be.

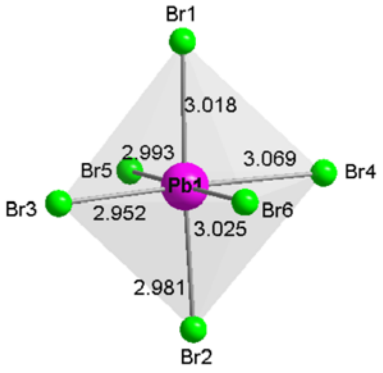

Figure S3. A distorted $PbBr_6$ octahedron

Herein, we can use three parameters, namely the tilting distortion parameter (Δ), the angle distortion parameter (Σ), and the off-center distortion parameter (Δ_{od}), to quantitatively compare the $PbBr_6$ octahedral distortions in our iso-BAPB, n-BAPB, and n-PAPB. Δ and Σ represent the distortion degree of the Pb-Br bond lengths and Br-Pb-Br cis-angles, respectively.[4] Δ_{od} describes the degree of deviation of the Pb atom from the ideal octahedral

center.[2] The calculation equations for Δ and Σ are listed below (Eq. S1 and Eq. S2). And the calculation method of Δ_{od} are also given in section.

$$\Delta = \sum_{i=1}^{6} \left(\frac{d_i - \langle D \rangle}{\langle D \rangle}\right)^2 \quad (S1)$$

$$\Sigma = \sum_{i=1}^{12} |90 - \varphi_i| \quad (S2)$$

where the d_i are the individual Pb-Br bond distances in a the $PbBr_6$ octahedron. <D> is the average Pb-Br bond length, The φ_i are the twelve cis-angles of Br-Pb-Br around the Pb atom.

For the calculation of Δ_{od}, we can first define three trans-bond angles based on Figure S3 are as follows: θ_1 = ∠Br1-Pb1-Br2, θ_2 = ∠Br3-Pb1-Br4, θ_3 = ∠Br5-Pb1-Br6. Then, taking the difference in the associated bond lengths and dividing by the cosine of each angle results in the magnitude of the Δ_{od}:[2]

$$\Delta_{od} = [|(Pb1\text{-}Br1) - (Pb2\text{-}Br2)| \div |\cos\theta_1|] + [|(Pb1\text{-}Br3) - (Pb1\text{-}Br4)| \div |\cos\theta_2|] + [|(Pb1\text{-}Br5) - (Pb\text{-}Br6)| \div |\cos\theta_3|] \quad (S3)$$

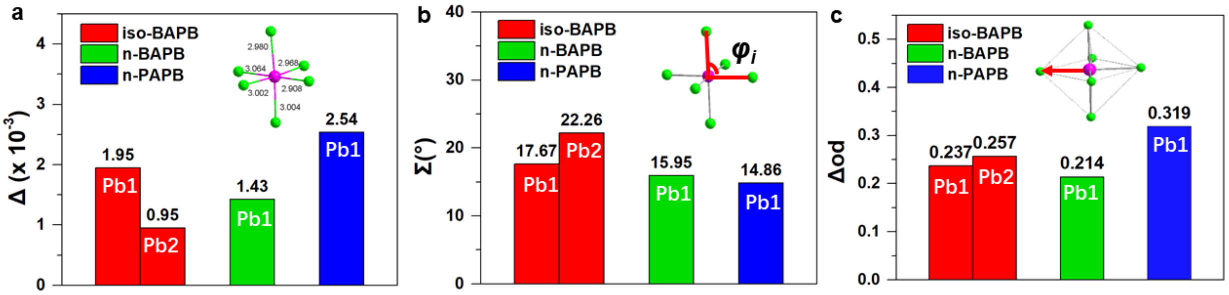

Figure S4 The distorted parameters of $PbBr_6$ octahedrons, tilting distortion parameter (Δ) (a), the angle distortion parameter (Σ) (b), and the off-center distortion parameter (Δ_{od}) (c).

The calculation results of these three parameters are shown in the Figure S4a-c. It is worth noting that there are two types of Pb^{2+} ions in iso-BAPB (i.e., Pb1 and Pb2) since it crystallizes in a low symmetry monoclinic space group. In this work, we took their average parameter value to represent its $PbBr_6$ distortion. The distortion analysis revealed that the Δ and Δ_{od} values of n-PAPB are much larger than that of iso-PAPB and n-BAPB. On the other hand, the value of Σ in iso-BAPB is the largest one (19.97°), while the values of n-BAPB (15.95°) and n-PAPB (14.86°) are relatively close. Overall, we can conclude that the $PbBr_6$ octahedron of n-BAPB is the least distorted, whereas that of n-PAPB is distorted the strongest to relax the interface lattice strain.

S3 Calculation of the lattice mismatch between our three 2D RP perovskites.

The lattice mismatches between our 2D RP perovskites and 3D $MAPbBr_3$ were calculated by the following equation:[5]

$$f = \frac{(\alpha_{2D} - \alpha_{3D})}{\alpha_{3D}} \times 100\% \qquad \text{(S4)}$$

where the α_{2D} is the lattice constant along the 2D layered facets, which were taken from the resolved structures by us (Table S1, Figure S5); α_{3D} is the lattice constant of 3D $MAPbBr_3$ (*I*4/*mcm*) (Figure S5a).[6] The calculation results of lattice mismatches were shown in the Figure S5e.

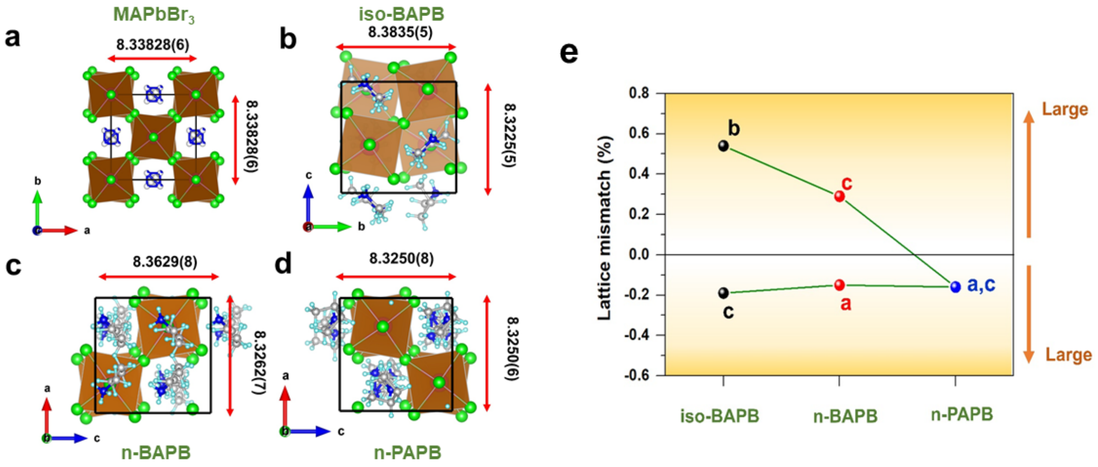

Figure S5, the lattice constants of 3D $MAPbBr_3$ (*I*4/*mcm*) (a), and the lattice constants along the 2D layered facets of iso-BAPB (b), *n*-BAPB (c), and *n*-PAPB (d); the lattice mismatch between the three 2D RP perovskites and 3D $MAPbBr_3$ (f).

S4 Calculations of the penetration depths and excitation density of the three compounds.

To calculate the penetration depths and excitation density of these samples, we first measured their absorption coefficients using the laser. In order to ensure that the laser spot is completely illuminated on the samples, we prepared a sufficiently large film according to the method in the literature.[7] After the absorption measurements, we used AFM to determine the thickness of the film (Figure S6). The absorbance (A) can be calculated by the following formula:

$$A = \log (I_0/I_1) \tag{S5}$$

Here, I_0 is incident light intensity and I_1 is outgoing light intensity. After measuring the A values at 375 nm of the iso-BAPB, n-BAPB and n-PAPB are about 0.37, 0.39 and 0.33, respectively. The absorption coefficient ε = 2.303A/l, where l is the path length of the light which equals to the thickness of the crystal. So, we obtain ε =1.40 × 10^5, 1.38 × 10^5 and 1.27 × 10^5 cm^{-1} for iso-BAPB, *n*-BAPB and *n*-PAPB, respectively. The penetration depths of them at 375 nm are about 71, 73 and 79 nm, respectively ($\delta = 1/\varepsilon$). The excitation density n can be calculated as photon flux f in photons/cm^2 multiplied by absorption coefficient ε: $n = f\varepsilon$, Table S4 summarizes the corresponding excitation density at different laser photon flux in TRPL measurements:

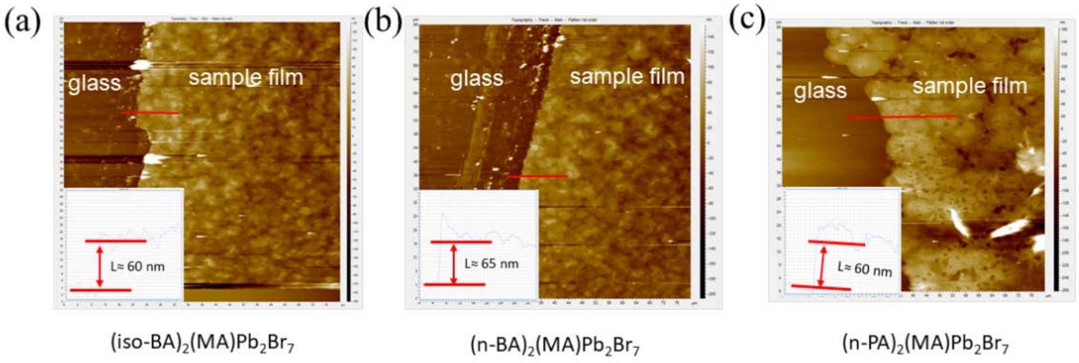

Figure S6**.** The AFM pictures of iso-BAPB (a), *n*-BAPB (b) and *n*-PAPB (c) films with the thickness values.

Table S4 Excitation density of iso-BAPB, *n*-BAPB and *n*-PAPB with different laser photon flux.

**Excitation Fluence (photon/pulse/cm^2)**	**Excitation Intensity (cm^{-3}) of iso-BAPB**	**Excitation Intensity (cm^{-3}) of *n*-BAPB**	**Excitation Intensity (cm^{-3}) of *n*-PAPB**
8.9 ×10^7	1.25 × 10^{13}	1.23 × 10^{13}	1.13 × 10^{13}
1.3 ×10^8	1.82 × 10^{13}	1.79 × 10^{13}	1.65 × 10^{13}
6.4 ×10^8	8.96 × 10^{13}	8.83 × 10^{13}	8.13 × 10^{13}
1.5 ×10^9	2.10 × 10^{14}	2.07 × 10^{14}	1.91 × 10^{14}
3.0 ×10^9	4.20 × 10^{14}	4.14 × 10^{14}	3.81 × 10^{14}

S5. Calculation of electron-phonon coupling strength using temperature dependent photoluminescence method.

PL spectra of these three compounds were measured at temperatures ranging 100-280 K in a cryostat (Figure S7), liquid nitrogen as the coolant. In order to verify our argumentation, we calculate the electron-phonon coupling strength from the FWHMs of the temperature-dependent PL spectra using the following moldel:[8,9]

$$\Gamma(\mathrm{T}) = \Gamma_0 + \Gamma_{\mathrm{ac}} + \Gamma_{\mathrm{LO}} + \Gamma_{\mathrm{imp}} = \Gamma_0 + \gamma_{ac}T + \gamma_{LO}N_{\mathrm{LO}}(T) + \gamma_{imp}e^{-E_b/k_BT} \quad \text{(S7)}$$

Here, Γ_0 is a temperature-independent inhomogeneous broadening that arises from scattering due to disorder and imperfections. Γ_{ac} and Γ_{LO} are homogeneous broadening terms, which result from acoustic and Fröhlich scattering, with charge-carrier phonon coupling strengths of acoustic phonon modes γ_{ac} and LO phonon modes γ_{LO}, respectively. N_{LO} (T) = $1/(e^{E_{LO}/k_BT}$ -1), where the E_{LO} is an energy representative of the frequency for the weakly dispersive LO phonon branch, and k_B is Boltzmann constant. Γ_{imp} is the inhomogeneous broadening due to the ionized impurities. It is worth noting that Γ_{ac} and Γ_{imp} do not contribute much to the temperature dependence at higher temperatures and they can be included to Γ_0.[8] Hence, we model the linewidth broadening of these three SCs using equation S8 (Figure S8).

$$\Gamma(\mathrm{T}) = \Gamma_0 + \gamma_{LO}N_{\mathrm{LO}}(T) \quad \text{(S8)}$$

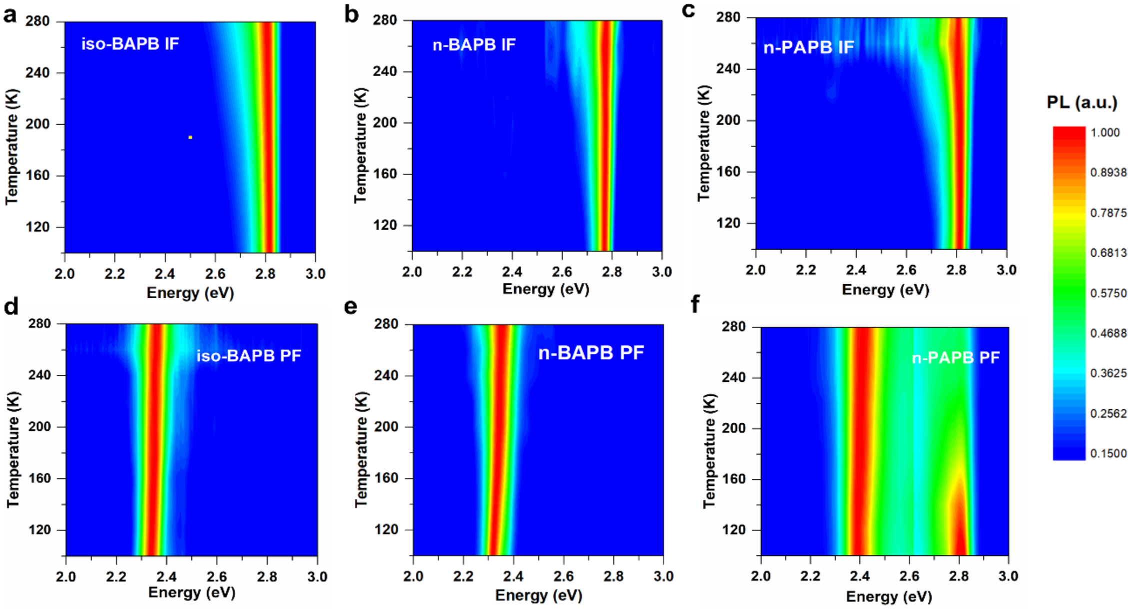

Figure S7 Temperature-dependent PL spectra of IFs and PFs of the three SCs.

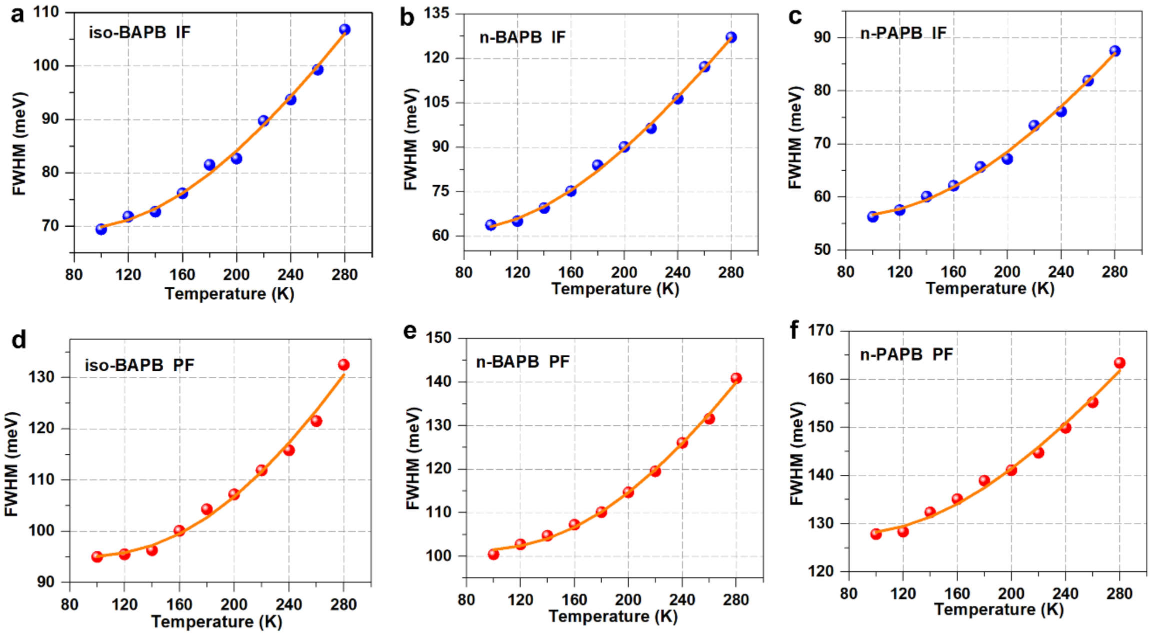

Figure S8**.** FWHM of the temperature-dependent PL for the IFs and PFs of the three SCs, and the solid orange lines fit equation S8 in main text.

S6. The fitting analysis of the PL decay.

We can fit the PL kinetics using triexponential decays with one fast component (t_1), a medium component (t_2), and a slow component (t_3) (Table S5). The slow components t_3 has a very small amplitude and can be neglected. Here t_1 and t_2 can be assigned to the trapping related nonradiative recombination and radiative recombination of the photoexcited charge carriers, respectively.

Table S5 Multi-exponential fitting parameters for TRPL kinetics of iso-BAPB, *n*-BAPB and *n*-PAPB under the excitation fluence of 1.3 × 10^8 photon/pulse/cm^2.

IFs	A_1(%)	t_1 (ns)	A_2(%)	t_2 (ns)	A_3(%)	t_3 (ns)
iso-BAPB	88.4	0.398 (±0.001)	8.2	8.15 (±0.02)	3.4	84.4 (±0.2)
n-BAPB	87.9	0.549 (±0.001)	9.8	7.29 (±0.03)	2.3	75.6 (±0.2)
n-PAPB	93.4	0.492 (±0.002)	5.6	5.98 (±0.03)	1.0	76.3 (±0.4)

PFs	A_1(%)	t_1 (ns)	A_2(%)	t_2 (ns)	A_3(%)	t_3 (ns)
iso-BAPB	84.1	0.481(±0.002)	13.6	5.05 (±0.02)	2.3	52.2(±0.2)

n-BAPB	68.0	1.736 (±0.006)	48.7	8.02 (±0.04)	3.3	50.7(±0.2)
n-PAPB	71.9	1.123 (±0.006)	23.7	10.24 (±0.02)	4.4	98.9 (±0.4)

S7. Detailed model of trap filling and fitting process.

The detailed calculation procedures of the model have been described in the reference.[10] In brief, the equilibrium between free carriers and excitons can be described using the Saha equation. When photodoping (i.e accumulated trap filling) is present, the Saha equation can be generalized as the following equation expressing the concentrations of electrons (n_e), holes (n_h) and excitons (n_x) corresponding to the overall untapped photogenerated species density N:

$$N_h = -\frac{(A - n_T)}{2} + \frac{1}{2}\sqrt{(A+N_T)^2 + 4AN} \quad \text{(S9)}$$

$$n_e = n_h - n_T \quad \text{(S10)}$$

where $A = v_x/(v_h v_e)\ exp[-E_b/(k_B T)]$ and $v_i = \lambda_i^3$, λ_i is the thermal wavelength of the species i. n_T is concentration of filled traps and N_T is the concentration of total traps.

Here we assume that n_T varies little and is only dependent on average concentration of electrons $\overline{n_e(t)}=\frac{1}{t_0}\int_0^{t_0} n_e(t)$ during the PL recording time t_0 among the repetition pulses. Then we can get the following rate equations:

$$\frac{dn_T}{dt} = R_{pop}(N_T - n_T)\overline{n_e(t)} - R_{dep}\left(N_T^2 + n_T\,\overline{n_e(t)}\right) = 0 \quad \text{(S11)}$$

where R_{pop} and R_{dep} are the recombination rates of trap population and depopulation, respectively. Taking equations (S9) and (S10) we can obtain the average concentration of electrons as:

$$\overline{n_e(t)} = \frac{1}{t_0}\int_0^{t_0} n_e(t) \approx K(A + n_T),\ K = \frac{1}{\gamma_0 t_0}\ln[1 + \frac{AN(0)}{N_T(A + N_T)}] \quad \text{(S12)}$$

Here $N(0)$ can be simplified as initial excitation density N_c. γ_0 is the total rate of electronic decay not involving traps. Substitution of equation (S11) into (S12) gives:

$$n_T = -\frac{1}{2}\alpha + \frac{1}{2}\sqrt{\alpha^2 + 4\beta N_T} \quad \text{(S13)}$$

$$\alpha = \frac{[A + R(A - N_T)]}{\left(1 + \frac{1}{K} + R\right)} \quad \text{(S14)}$$

$$\beta = \frac{RA}{\left(1 + \frac{1}{K} + R\right)} \quad \text{(S15)}$$

$$K = \frac{1}{\gamma_0 t_0}\ln\left[1 + \frac{AN_c}{N_T(A+N_T)}\right],\ R = R_{pop}/R_{dep} \quad \text{(S16)}$$

During the fitting of the trapping model, we first assume that both traps exhibit filling but due to different trap population and depopulation rates, the ratio of unoccupied trap densities between two type of traps varies with different excitation intensity (Figure S9a-b). Therefore we can plot $A_1/(A_1+A_2)$~N_c data and fit with the expression combination equations S13-S16.

$$\frac{A_1}{A_1+A_2}=\frac{n_{\mathrm{uncT1}}}{n_{\mathrm{uncT1}}+n_{\mathrm{uncT2}}}=\frac{N_{T1}+\frac{1}{2}\alpha_1-\frac{1}{2}\sqrt{\alpha_1^2+4\beta_1 N_{T1}}}{(N_{T1}+\frac{1}{2}\alpha_1-\frac{1}{2}\sqrt{\alpha_1^2+4\beta_1 N_{T1}})+(N_{T2}+\frac{1}{2}\alpha_2-\frac{1}{2}\sqrt{\alpha_2^2+4\beta_2 N_{T2}})} \qquad (S17)$$

Here n_{uncT1} and n_{uncT2} are the concentration of filled traps 1 and 2, respectively. N_{T1} and N_{T2} are the concentrations of original concentration of trap 1 and trap 2, respectively. In this work, A of iso-BAPB and *n*-BAPB were approximately equal to 0.97×10^{15} cm^{-3}, respectively, PL recording time t_0 was 2.5×10^{-7} s, and γ_0 was taken as the rate of the charge recombination not contributing to the trap filling process, we first set all the four fitting parameters (N_{T1}, N_{T2}, R_1, and R_2) while analyzing these two compounds. The best fitting results as shown in Table S6. In this table, R_2 of iso-BAPB and *n*-BAPB are 15.5 and 36.7, respectively. Considering R is the ratio between trap population and depopulation rates, we can then roughly estimate the depopulation time of their traps 2 using the trap population time obtained in PL kinetics (lifetime of the second component 5.05 and ~8.02 ns, respectively) to be ~80 and ~290 ns, respectively. These depopulation time of traps 2 are not much different from the interval between the pulses in our measurement (250 ns). Therefore, we conclude that no considerable trap filling should occur in their traps 2 since the depopulation rate is too fast.[11] The model is then modified for the case that trap filling occurs only in trap1 as follows:

$$\frac{A_1}{A_1+A_2}=\frac{n_{\mathrm{uncT1}}}{n_{\mathrm{uncT1}}+N_{T2}}=\frac{N_{T1}+\frac{1}{2}\alpha_1-\frac{1}{2}\sqrt{\alpha_1^2+4\beta_1 N_{T1}}}{(N_{T1}+\frac{1}{2}\alpha_1-\frac{1}{2}\sqrt{\alpha_1^2+4\beta_1 N_{T1}})+N_{T2}} \qquad (S18)$$

Here the $A_1/(A_1+A_2)$ represents the ratio between unoccupied density of trap 1 and original density of trap 2. We first set all the three fitting parameters (N_{T1}, N_{T2}, and R) free for the values from the equation (S17). Then, we fit the equation (S18) (Figure S9c) to get the new parameters (Table S6).

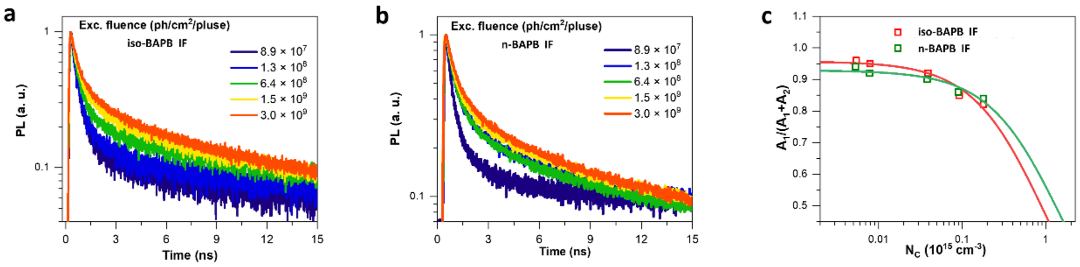

Figure S9. PL decay kinetics with different excitation fluence for the IFs of iso-BAPB (a) and *n*-BAPB (b); dependence of the amplitude ratios $A_1/(A_1 + A_2)$ in the multi-exponential fitting of the decays on the initial charge densities N_c for the IFs of iso-BAPB and *n*-BAPB (c).

Table S6. Trap Densities obtained from the fitting.

Model	IFs	R_1	R_2	N_{T1} (10^{15} cm^{-3})	N_{T2} (10^{15} cm^{-3})
$\frac{A_1}{A_1+A_2}=\frac{n_{uncT1}}{n_{uncT1}+n_{uncT2}}$	iso-BAPB	2.3×10^5	15.5	11.7	0.23
	n-BAPB	2.7×10^4	36.7	9.1	0.57
$\frac{A_1}{A_1+A_2}=\frac{n_{uncT1}}{n_{uncT1}+N_{T2}}$	iso-BAPB	1.4×10^5	/	46.9	2.08
	n-BAPB	5.1×10^4	/	29.1	2.19

S8. Calculation of the free carrier ratio after photo-excitation

After photoexcitation by the laser pulse, actually the free carriers and weak coupled excitons coexist in the crystal under a thermodynamics equilibrium. The ratio between two species is a fixed term resembling the ion-electron balance in a hot plasma, which depends strongly on the exciton binding energy as well as the excitation concentration. Therefore, we can use a classic Saha-Langmuir theory to roughly calculate the ratio of the free carriers X (i.e., fraction of the free carriers among all the excited species) in the system:

$$\frac{x^2}{1-x} = \frac{1}{n}\left(\frac{2\pi m k_B T}{h^2}\right)^{1.5} e^{-\frac{E_b}{k_B T}} \qquad \text{(S19)}$$

where E_b refers to the exciton binding energy (We obtained their E_b by fitting the temperature-dependent PL peak intensities of ~59 meV, ~93 meV, ~88 meV for iso-BAPB, *n*-BAPB, and *n*-PAPB, respectively),[4] m is the exciton effective mass, T is the temperature and *n* is the excitation concentration. Previous study has shown that this equation is also applicable to 2D hybrid lead halide perovskites.[12] The following table summarized the calculated x ratio of three samples under the excitation condition in PL decay measurement (Table S7). Apparently, during our measurement, the majority of the excited species in the 2D perovskite crystals is free carriers and the contribution of the exciton can almost be negligible.

Table S7. The calculated values of ratio of the free carriers x.

Samples	Excitation Intensity (cm^{-3})	x
iso-BAPB IF	$0.125 – 4.20 \times 10^{14}$	0.998 – 0.946

n-BAPB IF	0.123 – 4.14 × 10^{14}	0.993 – 0.839
n-PAPB IF	0.113 – 3.81 × 10^{14}	0.995 – 0.868

Table S8 Relative PLQY (%) at PFs of three samples (PL decay Aera/Exc. Fluence)

Samples / Exc. Fluence	iso-BAPB PF	*n*-BAPB PF	*n*-PAPB PF
8.9 × 10^7	0.00167	0.03288	0.01074
1.3 × 10^8	0.00154	0.02422	0.00984
6.4 × 10^8	0.00069	0.01062	0.00705
1.5 × 10^9	0.00065	0.00745	0.00672
3.0 × 10^9	0.00069	0.00687	0.00626

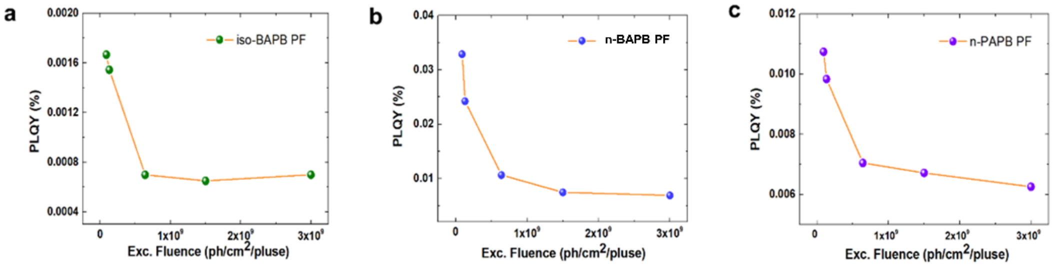

Figure S10. Relative PLQY (%) at PFs of three samples.

S9 Local strain accumulation and phonon coupling analysis of Br-based and I-based perovskites

First, the feature of the 'edge state' should be determined by the stiffness of the lattice. According to the theoretical calculation and the experimental measurement, the Br-based perovskites generally have a higher bulk modulus than I-based perovskites and become less prone to long-range lattice distortions.[13,14] This can lead to two consequences: 1) stronger strain accumulation in Br-based perovskite, 2) small instead of large polaron formation in Br-based perovskites corresponding to the short-range local lattice distortion (e.g. on the surface facet). In addition, the general e-LO phonon coupling strength can be calculated by the following equation:[15]

$$a = \frac{e^2}{h}\frac{1}{4\pi\epsilon_0}\sqrt{\frac{m^*}{2h\omega_{LO}}}\left[\frac{1}{\epsilon_\infty} - \frac{1}{\epsilon_s}\right] \tag{S20}$$

Here and ϵ_s and ϵ_∞ refers to the dielectric permittivities at static and optical frequencies, respectively. The term within the bracket evaluates the lattice polarity. The lattice polarity made by Pb-Br bonding should be higher than Pb-I due to the larger electronegativity difference between Pb and Br (0.7) compared with Pb and I (0.4). Therefore, we can expect high phonon coupling in Br-based system, which would facilitate the formation of polaron states. The above two reasons should encounter why in our Br-based 2D perovskites, the STE instead of conventional sub-gap states are formed at PFs.

Reference

(1) Liang, M.-L.; Ma, Y.-X.; Hu, C.-L.; Kong, F.; Mao, J.-G., $Ba(MoO_2F)_2(QO_3)_2$ (Q = Se, Te): Partial Fluorination of MoO_6 Octahedra Enabling Two Polar Solids with Strong and Phase Matchable SHG Response. *Chem. Mater.* **2020**, *32*, 9688-9695.

(2) Halasyamani. P. S. Asymmetric Cation Coordination in Oxide Materials:Influence of Lone-Pair Cations on the Intra-octahedral Distortion in d^0 Transition Metals. *Chem. Mater.* **2004**, *16*, 3586-3592.

(3) Zhu, Q.; Zheng, K.; Abdellah, M.; Generalov, A.; Haase, D.; Carlson, S.; Niu, Y.; Heimdal, J.; Engdahl, A.; Messing, M. E.; et al. Correlating Structure and Electronic Band-Edge Properties in Organolead Halide Perovskites Nanoparticles. *Phys. Chem. Chem. Phys.* **2016,** *18*, 14933-14940.

(4) Liang, M.; Lin, W.; Lan, Z.; Meng, J.; Zhao, Q.; Zou, X.; Castelli, I. E.; Pullerits, T.; Canton, S. E.; Zheng, K., Electronic Structure and Trap States of Two-Dimensional Ruddlesden–Popper Perovskites with the Relaxed Goldschmidt Tolerance Factor. *ACS Appli. Elect. Mater.* **2020,** *2*, 1402-1412.

(5) Stiff-Roberts. A. D. Quantum-Dot Infrared Photodetector. *Materials Science and Materials Engineering* **2011**, *6,* 452-485.

(6) López, C. A.; Martínez-Huerta, V. M.; Alvarez-Galván, M. C.; Kayser, Paula.; Gant, Pa.; Castellanos-Gomez, A.; Fernández-Díaz, M. T.; Fauth, F.;, Alonso, J. A. Elucidating the Methylammonium (MA) Conformation in $MAPbBr_3$ Perovskite with Application in Solar Cells. *Inorg. Chem.* **2017**, *56,* 14214–14219.

(7) Chen, Z.; Yu, C.; Shum, K.; Wang, J. J.; Pfenninger, W.; Vockic, N.; Midgley, J.; Kenney, J. T. Photoluminescence Study of Polycrystalline $CsSnI_3$ Thin Films: Determination of Exciton Binding

Energy. *J. Lumin.* **2012**, *132*, 345–349.

(8) Wright, A. D.; Verdi, C.; Milot, R. L.; Eperon, G. E.; Perez-Osorio, M. A.; Snaith, H. J.; Giustino, F.; Johnston, M. B.; Herz, L. M., Electron-Phonon Coupling in Hybrid Lead Halide Perovskites. *Nat. Commun.* **2016,** *7*, 11755.

(9) Shi, Q.; Ghosh, S.; Kumar, P.; Folkers, L. C.; Pal, S. K.; Pullerits, T.; Karki, K. J. Variations in the Composition of the Phases Lead to the Differences in the Optoelectronic Properties of $MAPbBr_3$ Thin Films and Crystals. *J. Phys. Chem. C* **2018**, 122 (38), 21817-21823.

(10) Stranks, S. D.; Burlakov, V. M.; Leijtens, T.; Ball, J. M.; Goriely, A.; Snaith, H. J. Recombination Kinetics in Organic-Inorganic Perovskites: Excitons, Free Charge, and Subgap States. *Phys. Rev. Appl.* **2014**. *2*, 034007.

(11) Zheng, K.; Žídek, K.; Abdellah, M.; Messing, M. E.; Al-Marri, M. J.; Pullerits, T. Trap States and Their Dynamics in Organometal Halide Perovskite Nanoparticles and Bulk Crystals. *J. Phys. Chem. C* **2016**, *120*, 3077–3084.

(12) Gelvez-Rueda, M. C.; Hutter, E. M.; Cao, D. H.; Renaud, N.; Stoumpos, C. C.; Hupp, J. T.; Savenije, T. J.; Kanatzidis, M. G.; Grozema, F. C. Interconversion between Free Charges and Bound Excitons in 2D Hybrid Lead Halide Perovskites. *J. Phys. Chem. C* **2017**, *121*, 26566−26574.

(13) Neukirch, A. J.; Abate, I. I.; Zhou, L.; Nie, W.; Tsai, H.; Pedesseau, L.; Even, J.; Crochet, J. J.; Mohite, A. D.; Katan, C.; Tretiak S. Geometry Distortion and Small Polaron Binding Energy Changes with Ionic Substitution in Halide Perovskites. *J. Phys. Chem. Lett.* **2018**, *9*, 7130–7136.

(14) Ferreira, A. C.; Létoublon, A.; Paofai, S.; Raymond, S.; Ecolivet, C.; Rufflé, B.; Cordier, S.; Katan, C.; Saidaminov, M. I.; Zhumekenov, A. A.; et al. Elastic Softness of Hybrid Lead Halide Perovskites. *Phys. Rev. Lett.* **2018**, *121*, 085502.

(15) Fu, J.; Xu, Q.; Han, G.; Wu Bo.; Huan, C. H. A.; Leek, M. L.; Sum, T. C. Hot carrier cooling mechanisms in halide perovskites. *Nat. Commun.* **2017**, *8*, 1300.
